# A Canine Leptospirosis Clinical Case Due to *Leptospira interrogans* (Serogroup Icterohaemorrhagiae) in a Dog Kennel in Castelvetrano (Western Sicily, South Italy)

**DOI:** 10.3390/vetsci10080508

**Published:** 2023-08-06

**Authors:** Francesca Grippi, Valeria Blanda, Paola Galluzzo, Manuel Bongiorno, Carmela Sciacca, Francesca Arcuri, Rosalia D’Agostino, Ilenia Giacchino, Francesca Gucciardi, Mario D’Incau, Cristina Bertasio, Alessandra Torina, Annalisa Guercio

**Affiliations:** 1 Istituto Zooprofilattico Sperimentale della Sicilia, Via Gino Marinuzzi 3, 90129 Palermo, Italy; francesca.grippi@izssicilia.it (F.G.); carmela.sciacca@gmail.com (C.S.); francescaarcuri11@gmail.com (F.A.); rosalia.dagostino1@gmail.com (R.D.); ileniajak2@gmail.com (I.G.); francesca.gucciardi@izssicilia.it (F.G.); alessandra.torina@gmail.com (A.T.); annalisa.guercio@izssicilia.it (A.G.); 2 Dipartimento di Prevenzione Veterinaria-Area di Sanità Pubblica Veterinaria, Unità Operativa Territoriale Alcamo Castelvetrano, Via Sardegna, 91022 Castelvetrano, Italy; manuelbongiorno@tiscali.it; 3 Istituto Zooprofilattico Sperimentale della Lombardia e dell’Emilia-Romagna, 25124 Brescia, Italy; mario.dincau@izsler.it (M.D.); cristina.bertasio@izsler.it (C.B.)

**Keywords:** dog, kennel, leptospirosis, zoonosis, MAT, molecular typing

## Abstract

**Simple Summary:**

Leptospirosis is a zoonosis caused by *Leptospira*, helical-shaped pathogenic spirochetes. *Leptospira* can affect both domestic and wild animals and humans, with clinical manifestations ranging from severe conditions to mild feverish symptoms or asymptomatic conditions. Leptospirosis is an occupational disease often overlooked in humans, possibly due to underdiagnosis, particularly in the absence of specific clinical signs. Its transmission can occur by direct contact with contaminated urine or organs. Dogs are reservoirs of pathogenic *Leptospira*, in particular *L. interrogans* serogroup Canicola, capable of infecting humans and other mammals. We report a case of acute leptospirosis in a puppy dog housed in a municipal kennel and the subsequent diagnostic investigations carried out on all the dogs housed in the kennel. The puppy tested positive for *Leptospira interrogans* serogroup Icterohaemorrhagiae in both serological and molecular tests. All of the other 66 dogs in the kennel underwent clinical and laboratory tests twice, 15 days apart. At the first sampling, eight dogs (12%) showed antibodies against *Leptospira interrogans* serogroup Icterohaemorragiae serovar Copenhageni and in one of them *Leptospira* spp. DNA was detected. Dogs, and kennel dogs in particular, are sentinels for many zoonotic diseases, likely due to high population densities, stress, and exposure to rodents and other disease vectors.

**Abstract:**

Leptospirosis is a worldwide widespread zoonosis caused by *Leptospira* genus. We report an acute leptospirosis case in a puppy housed at a municipal kennel and the subsequent diagnostic investigations carried out on all dogs housed in the kennel. Laboratory investigation included mainly a microagglutination test, real-time PCR, and multi-locus sequence typing (MLST) for *Leptospira* genus. Other agents of infection were excluded. The puppy resulted positive for *Leptospira interrogans* Icterohaemorrhagiae both with serological and molecular assays. All of the other 66 dogs in the kennel underwent clinical and laboratory investigations twice, 15 days apart. No other dog showed leptospirosis clinical signs. At the first sampling, eight dogs (12%) showed antibodies against *Leptospira interrogans* serogroup Icterohaemorragiae serovar Copenhageni. Real-time PCR on urine samples of seropositive dogs detected *Leptospira* spp. DNA in one sample, then identified as *Leptospira interrogans* serogroup Icterohaemorragiae by MLST. Fifteen days after, four of the previous seropositive dogs still showed antibodies against *Leptospira* spp. All urine samples collected from seropositive dogs were negative at real-time PCR. The study allowed the early confirmation of a Leptospirosis case and the identification of at least one asymptomatic carrier of pathogenic *Leptospira* spp. The prompt activation of all appropriate management measures allowed limiting and extinguishing the infection.

## 1. Introduction

Leptospirosis is a zoonosis spread worldwide [1,2] caused by the *Leptospira* genus (family *Leptospiraceae*, order *Spirochaetales*), pathogenic helical shaped spirochetes. Several species of both domestic and wild animals, as well as humans [3,4,5,6,7,8,9], can be affected by *Leptospira*. Clinical manifestations ranging from severe conditions to mild febrile symptoms or even asymptomatic conditions can be observed [10,11]. Its transmission may occur via direct contact with contaminated urine or organs, due to the bacterium ability of colonizing kidneys after the bacteraemic phase and being released in the urine [12], or indirectly through exposure to contaminated environments [13]. Environmental factors, such as high pluviometric precipitation rates, flooding, natural disasters, uncontrolled urban expansion, and poor sanitation, have a significant role in influencing its transmission [10,14]. Human leptospirosis is considered a neglected infectious disease worldwide, probably due to the failure in leptospirosis diagnosis, in particular in the absence of specific clinical signs [15,16,17]. Leptospirosis is considered an occupational disease for several worker categories [18,19,20] and, in the last few years, leptospirosis transmission due to recreational activities has emerged [18].

Dogs are reservoirs for pathogenic *Leptospira* [10,21], especially *L. interrogans* serogroup Canicola, able to infect humans and other mammals [22]. Infected dogs may show serious clinical outcomes, with acute hepatorenal failure, or also be asymptomatic chronic carriers [23,24]. The last ones are of public health concern as they may spread the infection. Kennel dogs, in particular, are sentinels for many zoonotic diseases, likely due to high population density, stress, and exposure to rodents and other vectors of diseases [25]. In Italy, a national serological surveillance of Leptospirosis carried out on 3028 dogs in the years 2010–2011 detected a positive reaction in 29.9% of the animals. Serovars Bratislava (serogroup Australis), Copenhageni (serogroup Icterohaemorrhagiae), Icterohaemorrhagiae (serogroup Icterohaemorrhagiae) and Canicola (serogroup Canicola) were the most frequently detected [26]. In particular, leptospiral infection was more common in dogs housed in kennels, with serological prevalence values ranging from 13.8% up to 49.2%, and strictly correlated with the hygienic conditions in the kennel [27].

According to the Italian Legislation, leptospirosis is ranked among the infectious and diffusive diseases and any confirmed or suspected case needs to be reported (Veterinary Police Regulation, articles n. 1, 2, and 10 of the Italian Presidential Decree n. 320, 8 February 1954). Appropriate control measures include the closure of the kennel and the isolation of the infected animals. Kennel closure is solved only with the clinical recovery following an adequate antibiotic therapy (Italian Ministerial Ordinance, 4 September 1985, article n. 6, specifically addressed to dogs and horses).

In this manuscript we describe the diagnostic path followed in order to detect the *Leptospira* presence and spread in a Sicilian municipal dog kennel (South Italy). The diagnostic process involved two phases: (i) the diagnosis of pathogenic *Leptospira* in a puppy dog that later died; (ii) the infection monitoring in the other dogs living in the kennel.

## 2. Materials and Methods

### 2.1. The Kennel

The kennel where the clinical case occurred is located in a countryside area in Castelvetrano, in Trapani province (Western Sicily, South Italy). A water purifier in the surrounding area was identified as a possible contributing factor for *Leptospira* spp. spreading. Moreover, evidence of rodent activity had been reported over time, and periodic rodent disinfections were carried out at the kennel. The kennel housed sixty-seven dogs, which underwent a medical examination at the time of entry into the kennel. As regards the vaccination status of the animals against *Leptospira* spp., all the dogs at the shelter were not vaccinated or vaccinated more than one year before with commercial tetravalent vaccine Nobivac L4 including *L. interrogans* serogroup Canicola, *L interrogans* serogroup Icterohaemorrhagiae, *L. interrogans* serogroup Australis, and *L. kirscheri* serogrop Grippotyphosa. No booster shots had been carried out with the recommended regularity. Table 1 shows the historical details of the vaccination status of all 67 dogs at the time of the clinical case occurring. These dogs were divided according to their vaccination status into three groups: Group A, with dogs never vaccinated, including the deceased puppy, Group B, including dogs whose vaccination was carried out between 1 and 2 years ago, and Group C, with animals vaccinated more than two years ago.

### 2.2. Samples

#### 2.2.1. Suspected Clinical Case

On the day of the onset of symptoms (T0), blood samples from the sick puppy were withdrawn and blood cells were separated from serum by centrifugation and stored at +4 °C until processing. At the same time, a urine sample was collected by cystocentesis and stored at +4 °C until the analysis. Following the animal’s death, an anatomopathological examination was carried out and a fragment of the kidney was collected and stored at +4 °C for immediate molecular analysis.

#### 2.2.2. Sampling from the Other Dogs Housed in the Kennel

At T0, blood samples were withdrawn from all of the other sixty-six dogs in the kennel, and blood cells were separated from serum by centrifugation. All the dogs were subjected to a second blood sampling 15 days after (T1). Serum samples were stored at +4 °C until analysis. Urine samples were collected by cystocentesis from seropositive dogs 4 days after T0 (T0 + 4d) and at T1 and stored at +4 °C until the analysis.

### 2.3. Sample Processing and DNA Extraction

The kidney surface was flamed and 1 g of tissue was withdrawn and homogenized in 9 mL of sterile physiological solution with a Stomacher^®^ 80 Biomaster (Seward Limited, London, UK) in order to perform DNA extraction. Two milliliters of urines were centrifuged at the rate of 12,000× *g* for 30 min, and the pellet was resuspended with 0.2 mL of ultrapure distilled water.

DNA was extracted from 0.2 mL of whole blood, homogenized kidney, or a resuspended pellet from urine using the Purelink Genomic DNA Kit (ThermoFisher Scientific, Rome, Italy) according to manufacturer’s instructions. For each sample, the extraction internal control (IC) included in the Quantifast Pathogen + IC Kit (Qiagen, Hilden, Germany) was used.

### 2.4. Differential Diagnosis

Real-time PCRs were carried out on the DNA extracted from the kidney fragment of the sick dog to detect DNA from *Chlamydia* spp. [28], *Neospora caninum* [29], *Coxiella burnetii* [30], *Borrelia burgdorferi* [31,32], and *Toxoplasma gondii* [33]. A nested PCR for *T. gondii* was also performed [34]. Regarding viral etiologic agents, conventional PCRs were carried out to detect Adenovirus [35] and Parvovirus [36,37] DNA.

### 2.5. Serological Test for Leptospirosis

Antibodies against *Leptospira* spp. were detected by a microagglutination test (MAT) in serum samples [38]. The MAT was performed according to the World Organization for Animal Health (WOAH) guidelines [39]. Cultured *Leptospira* spp. strains belonging to each of the eight pathogenic serogroups circulating in Italy were used in the test for sample agglutination, while specific *Leptospira* spp. rat antisera, provided by the Academic Medical Center (Amsterdam, the Netherlands), were used as positive controls. The following serovars were used as antigens: *L. interrogans* serogroup Australis, serovar Bratislava, strain Riccio 2; *L. borgpeterseni*, serogroup Ballum, serovar Ballum strain Mus 127; *L. interrogans*, serogroup Canicola, serovar Canicola, strain Alarik; *L. kirschneri*, serogroup Grippotyphosa, serovar Gryppotyphosa strain Duyster; *L. interrogans*, serogroup Icterohaemorrhagiae, serovar Copenhageni, strain Wjinberg; *L. interrogans*, serogroup Pomona, serovar Pomona, strain Pomona; *L. interrogans*, serogroup Sejroe, serovar Hardjo, strain Hardjoprajitno; and *L. borgpeterseni*, serogroup Tarassovi, serovar Tarassovi, strain Mitis Johnson.

Each antigen was supplied by the National Centre for Leptospirosis IZS of Lombardia and Emilia Romagna “Bruno Ubertini” (IZSLER, Brescia, Italy) or by the Amsterdam University Medical Centers. They were maintained in Leptospira Medium Base Ellinghausen–MacCullough–Johnson–Harris (EMJH—Difco, Becton, Dickinson, and Company, Sparks, MD, USA), subcultured every 7–10 days, and checked for purity, mobility, and agglutination power before use [38,40]. A MAT cut-off of 1:100 identified the serological positive samples. Two-fold serial dilutions from 1:100 up to 1:6400 were performed in order to evaluate the titer of positive sera. If a simultaneous reaction with different serogroups/serovars was observed, the strain determining the highest titer is reported in this study as the agent responsible for the serological positivity. If no differences were observed in titers, each of the serogroups/serovars could be responsible for the serological positivity.

### 2.6. Molecular Tests for Leptospirosis

A real-time PCR targeting the *lipL32* gene, which encodes an outer membrane protein present solely in the pathogenic species [41], was performed. DNA extracted from cultured *Leptospira interrogans* serogroup Australis serovar Bratislava was used as a positive control. The reaction was carried out in a CFX96 real-time system (Biorad, Segrate, Italy), applying a previously reported thermal profile [42].

### 2.7. Multi-Locus Sequence Typing (MLST)

In order to genotype the strain of *Leptospira* present in the samples, MLST assay was carried out on DNA from the dog samples that showed positive results in the real-time PCR. The MLST was carried out by the Italian National Reference Centre for Leptospirosis, at the Istituto Zooprofilattico Sperimentale della Lombardia e dell’Emilia Romagna, Brescia (Italy) [43,44,45,46]. Seven housekeeping genes were sequenced: *glm*U, *pnt*A, *suc*A, *tpi*A, *pfk*B, *mre*A, and *cai*B, based on scheme 1 proposed by Boonsilp in 2013 [44,47]. Nucleotide sequences were assembled using the SeqMan module of the Lasergene sequencing analysis software package (DNASTAR Inc., Madison, WI, USA) or using Bionumerics software ver. 7.6 (Applied Math, Biomerieux, Sint-Martens-Latem, Belgium). For allelic number and ST identification, assembled and trimmed sequences were queried against the Bacterial Isolate Genome Sequence Database (BIGSdb) (available online: https://pubmlst.org/Leptospira/, accessed on 15 May 2022). A phylogenetic analysis was conducted on the concatemers of the seven MLST genes using MEGA X [48], by comparing the sequences obtained in this study and some reference sequences downloaded from BIGSdb that were related to the most common STs found in Italy.

### 2.8. Prevention Measures at the Kennel

Following the confirmation of leptospirosis in the clinical case, the kennel was temporarily closed, and a microchip was inserted in all the dogs. Both the incoming and outgoing movements of the animals were temporarily blocked, as well as the dog walking time. Immediate and extraordinary disinfection and rodent infestation control of all the boxes and surrounding areas were carried out. Appropriate communications were addressed to all the people working with dogs, in order to let them know about their sanitary conditions and to instruct them on the preventive measures to be taken and the use of personal protective equipment. A second round of disinfection and rodent control in the municipal dog kennel was carried out close to the first one.

### 2.9. Ethical Statement

All methods were carried out in accordance with relevant guidelines and regulations. This study was conducted as part of the IZS SI 09/15 RC research project approved by the Italian Ministry of Health on 29 July 2016 (DGSAF-0018379-P-29/07/2016) and by the Institutional Review Board of Istituto Zooprofilattico Sperimentale della Sicilia “Adelmo Mirri” (protocol code U/0013035, 14 September 2016).

Biological samples were taken from animals suspected of leptospiral infection, and the sampling was necessary in order to perform laboratory analysis. The study did not involve any suffering of the animals sampled.

## 3. Results

### 3.1. Leptospirosis Clinical Case: From Clinical Suspicion to Laboratory Confirmation

Here, we report a case of canine leptospirosis that occurred in 2019 at the municipal dog kennel of Castelvetrano (South Italy). The suspicion of leptospirosis concerned a two-month-old mestizo puppy that was born in the shelter. The sick puppy dog stopped eating on the first day, reporting mild hyperthermia and dehydration. Jaundice was noticed on the second day. The puppy had not been vaccinated against *Leptospira* spp. (Group A of Table 1).

Serological test for leptospirosis carried out on the serum of the sick puppy showed antibodies against *L. interrogans* serogroup Icterohaemorragiae serovar Copenhageni (titer of 400). Also, a reaction against *L. interrogans* serogroup Grippotyphosa serovar Grippotyphosa was observed (titer of 200). Urine samples were found to be negative for pathogenic *Leptospira* spp. DNA.

Doxycycline antibiotic was administered only for a day as the animal’s condition worsened and it died on the third day. Postmortem anatomopathological examination reported a yellow and black coloration of cutis and digital bearings, presence of intestinal intussusception. Molecular analysis carried out on the kidney fragment revealed the presence of pathogenic *Leptospira* spp. DNA, with Ct = 34.2. All of the other molecular tests carried out on the kidney sample for differential diagnosis showed negative results.

### 3.2. Investigations on the Other Dogs in the Kennel and Containment Measures

Four of the sixty-six dogs housed in the shelter lived in the same box as the dead puppy. They were monitored throughout the study, and none of them showed leptospirosis clinical signs, and nor did the other animals in the kennel. All the animals housed in the shelter underwent serological analyses. At the first check (T0), eight out of sixty-six sera samples were found to be positive (12%) (Table 2) for antibodies to *L. interrogans* serogroup Icterohaemorragiae serovar Copenhageni, with titers of 1600, 400, 200, and 1100 (Table 3). The dog n. 6 showed the same titer of antibodies (100) both against *L. interrogans* serogroup Icterohaemorragiae serovar Copenhageni and *L. interrogans* serogroup Australis serovar Bratislava.

In addition, the urine sample (T0 + 4d) of the dog with the highest antibody titer (dog n. 1) against the pathogen was positive for *Leptospira* spp. DNA (Ct = 35.8). As a precaution, all the seropositive dogs as well as the four dogs living with the sick puppy were subjected to antibiotic therapy. The antibiotic treatment consisted of a 5 mg/kg dose of doxycycline orally repeated every 12 h for a period of 15 days [12].

All the animals were subjected to a second serological control 15 days after (T1). Three out of the previous positive dogs showed the same antibody titer against *Leptospira* spp., one animal had a decreased titer, while the other four dogs were negative. None of the other animals was found to be positive at the second serological survey.

All the urine samples collected at T1, after the antibiotic treatment, from serological positive dogs showed negative for the detection of pathogenic *Leptospira* spp. DNA, including the dog n. 1, despite its high antibody titer.

Concerning their vaccination status, dogs from n. 1 to n. 5 belonged to Group A, including subjects never vaccinated (Table 1 and Table 3); dogs n. 6, n. 7, and n. 8 belonged to the Group B, including animals vaccinated between one and two years (Table 1 and Table 3).

### 3.3. Genotyping Analyses

Both the kidney sample of the puppy and the urine sample of the dog n. 1 were subjected to genotyping analyses. The complete MLST profiles obtained from the samples are reported in Table 4.

In both cases, detected *Leptospira* belonged to ST17 that clustered with reference strains characterized as *L. interrogans* (serogroup Icterohaemorrhagiae) from the PubMLST and Italian Reference Center for Animal Leptospirosis (IZSLER, Brescia, Italy) databases.

**Figure 1:**
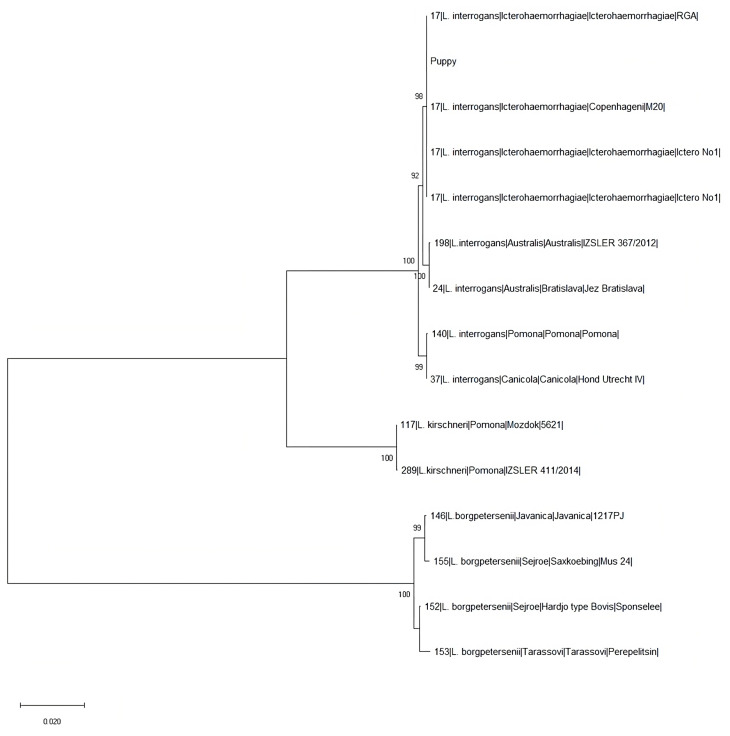
Phylogenetic tree built on concatenated sequences of the seven multi-locus sequence typing (MLST) loci (3111 bp) of scheme 1. Phylogeny was conducted in MEGA X using the Neighbor-Joining method and bootstrap values are indicated on the respective branches. The samples are indicated with ST, species, serogroup, serovar, strain.

Although we were not able to obtain a complete profile for the urine sample of dog n. 1, probably due to the low concentration or poor quality of leptospiral DNA in the sample (Ct = 35.8), in both cases, detected *Leptospira* undoubtedly belonged to ST17 that clustered with reference strains characterized as *L. interrogans* (serogroup Icterohaemorrhagiae) from the PubMLST and Italian Reference Center for Animal Leptospirosis (IZSLER, Brescia, Italy) databases (Figure 1).

## 4. Discussion

This study reports the occurrence of a leptospirosis clinical case in a puppy housed in the municipal kennel in Castelvetrano (Trapani, western Sicily, southern Italy). As previously reported, stray dog populations and dogs kept under kennel conditions may show a higher environmental exposure to pathogenic *Leptospira* spp. [10]. Following clinical suspicion, laboratory analyses allowed us to confirm a leptospirosis case in the sick puppy.

Although the urine sample collected from this animal was negative for *Leptospira* spp. DNA, this is consistent with our previous unpublished data and with results obtained from other studies reporting an intermittent release of bacteria in urine that is more evident in dogs with a lower antibody titer [49,50]. Serological analyses as well as molecular investigation carried out on a kidney fragment confirmed positivity for pathogenic *Leptospira* spp. Molecular typing allowed us to identify ST17, referred to as *L. interrogans* serogroup Icterohaemorrhagiae.

Promptly, all the dogs housed in the shelter underwent both clinical and laboratory investigations twice 15 days apart. Eight dogs were found to be seropositive to *L. interrogans* serogroup Icterohaemorragiae serovar Copenhageni at the first sampling. At the second survey, four of these dogs were still positive, and the antibody titers of these animals remained constant or decreased, probably thanks to the administered antibiotic therapy. No other animals showed clinical signs consistent with leptospirosis infections. All the seropositive samples reacted against the Copenhageni serovar. This pathogen has been related to severe leptospirosis in humans [51].

This is not the first description of an outbreak in a kennel on the island. In 2013, an outbreak was recorded in Palermo municipal kennel. Also in that case, the outbreak identification followed the sudden death of a dog housed in the kennel. However, unlike this study, the dog arrived at the kennel only a few days earlier already in poor health, and did not have contact with other housed dogs. Nevertheless, the kennel was closed, and all the 413 housed dogs underwent serological screening. Of these, 47 dogs showed positive in MAT testing and were promptly subjected to antibiotic therapy. Furthermore, as in the case described here, repeated disinfestations and rodent control were carried out. After the antibiotic treatment, a second serological control was carried out, and 23 of the positive dogs became negative, while the others either had negative seroconversion or maintained a constant antibody titer, leading to the outbreak resolution. Differently from our study, the authors hypothesized that the two events (the dog death and serological positivity of the dogs housed in the kennel) were independent [52].

In another study carried out in North Italy, the authors reported an outbreak case in a kennel with 3 dogs showing clinical manifestations attributable to leptospirosis. Furthermore, 50.8% of dogs living there (apparently healthily) were serologically positive in MAT and/or molecular investigations, indicative of *Leptospira* infection belonging to the Sejroe serogroup. Since positive dogs also included subjects who had never come into contact with each other, the data suggested that the *Leptospira* spread was caused by the circulation of infected rodents [53].

Serological results obtained in this study are in agreement with our previous findings of Serovars Copenhageni, Bratislava, and Canicola in Sicilian stray dogs [38]. A recent survey carried out in the other major Italian island, Sardinia, reported that 13% (164/1289) of dogs were sero-reactive in the MAT test. Specifically, the prevalence rate was 89% in kennel dogs and 11% in owned dog. Five *Leptospira* serovars were detected: serovar Bratislava, Icterohaemorrhagiae, and Copenhageni were prevalent, while Canicola and Grippotyphosa were also found [54].

An epidemiological survey carried out in Europe and based on serological analyses reported both the Icterohaemorrhagiae and Copenhageni serotypes as highly prevalent in dogs, reporting prevalence values up to 20% in some countries [55,56]. Other previous studies reported *L. interrogans* serogroup icterohaemorrhagiae as the major cause of canine leptospirosis in Europe, with prevalence values of even up to 20% in some countries [55,56]. Interestingly, this pathogen has been related to severe leptospirosis in humans [51].

Unfortunately, since the absence of an isolated strain on which to conduct a serological test with specific serovar monoclonal antibodies, as shown in Figure 1 it is not possible to discriminate between serovar Icterohaemorrhagiae and Copenhageni basing on MLST genotyping results, due to the very similar genetic content of these two serovars [57,58]. In our study, three dogs (dogs n. 1, n. 2 and n. 6) showed cross-reactions among different serovars, an effect caused by antigenic similarity. This aspecific reaction, well known among experts, represents one of the limits of MAT, especially in the early stages of the infection [59].

Among the seropositive animals, the urine of one dog tested positive at real-time PCR. This is an important aspect not only at individual level, for the diagnosis and to direct the therapeutic choice, but also from an epidemiological point of view, since we can state that at least one (dog n. 1) was able to release the bacteria into the environment, acting as a healthy eliminator and a carrier of pathogenic *Leptospira* spp. The genotyping analysis on dog n. 1 also confirmed the same ST obtained from the puppy dog, as expected. The early diagnosis, together with the containment measures promptly implemented in the kennel, made it possible to contain the spread of the infection within the kennel. Early identification of dogs with *Leptospira* spp. infection is, thus, also of primary importance because humans may be infected by direct or indirect contact with infected animals and their urine or by contact with contaminated water and soil [60]. In Italy, few recent data are available on human leptospirosis. However, other studies have shown the prevalence of infection in risk categories (hunters, farmers, forest workers, veterinarians, miners, rice field workers, soldiers, slaughterhouse workers) [61,62] and in the categories involved in outdoor or recreational activities, such as swimmers, campers, and rafters [63]. Unfortunately, in the absence of symptoms, laboratory investigations for leptospirosis are not frequently carried out.

The MAT panel used in this study included the eight serogroups circulating in Italy. This laboratory choice had the limitation of not detecting potential new circulating species, even if in this case the serological positivity was also confirmed by molecular typing by MLST.

Vaccination against leptospirosis has contributed to the decrease in infection by *Leptospira interrogans* serogroup Canicola serovar Canicola in Europe [64], including Italy [26,27]. A tetravalent vaccine (*L. interrogans* serogroup Canicola, *L interrogans* serogroup Icterohaemorrhagiae, *L. interrogans* serogroup Australis, *L. kirscheri* serogrop Grippotyphosa) is now available against the four species that commonly infect dogs [38,64,65]. It should also be noted that the *Leptospira* serogroups highlighted up to now in Sicily [38] are among those against which the available tetravalent vaccines protect.

Most of the dogs at the shelter were either unvaccinated or had been vaccinated more than a year prior to cases of infection. Unfortunately, MAT does not allow us to discriminate the antibodies titres deriving from vaccination or from natural infection, nor to obtain precise information about the time when the infection occurred [27]. Therefore, it is not possible to exclude the idea that the detected antibodies in some of the serological positive dogs (presumably animals n. 6, n. 7, and n. 8) had been stimulated during the previous vaccination. Indeed, even if antibody titers decreased or could no longer be detected one year after vaccination, it was reported that *Leptospira*-vaccinated dogs were still protected from clinical disease, infection, and urinary excretion [66]. In the case of the puppy, on the other hand, we can hypothesize that the serious disease was also correlated and facilitated by its young age associated to its non-vaccination status. Furthermore, it has been reported that the various boosters given at least annually enhance the effectiveness of the T cell-mediated immune response and increase specific IgG antibody production [65]. Another limitation of the study is the use of a MAT panel including only the eight serogroups circulating in Italy.

## 5. Conclusions

The obtained results allowed us to identify a clinical case of leptospirosis and at least one asymptomatic carrier both due to *L. interrogans* serogroup Icterohaemorragiae. The asymptomatic dog could contribute to the spread of the infection in the absence of early diagnosis and timely containment measures activated. Early diagnosis combined with the implementation of containment strategies, such as standardized hygiene protocols, rodent infestation control, and proper management of asymptotically infected dogs, has resulted in the effective prevention of the spread of infection. The abovementioned measures, in association with vaccination campaigns and annual vaccination boosters, are crucial for limiting the circulation and diffusion of *Leptospira* spp. and for safeguarding the health of dogs.

## Figures and Tables

**Table 1:** Vaccination status against *Leptospira* spp. of the dogs housed in the kennel.

Group	Vaccination Status	Number of Dogs
A	Never vaccinated	42/67
B	Vaccination between 1 and 2 years ago	12/67
C	Vaccinated more than 2 years ago	13/67

**Table 2:** Serum samples of 66 dogs by microscopic agglutination test (MAT).

Time	Negative Samples (%)	Positive Samples (%)
T0	58/66 (87.9%)	8/66 (12.1%)
T1	62/66 (93.9%)	4/66 (6.1%)

**Table 3:** *Leptospira* spp. serogroup/serovar and antibody titers of the positive serum samples.

ID Positive Sample (*n* = 66)	*Leptospira* spp. Serogroup/Serovar	Titer T0	Titer T1	Vaccination Status
Dog n. 1	*L. interrogans* serogroup Icterohaemorragiae serovar Copenhageni	1600	1600	
	*L. interrogans* serogroup Australis serovar Bratislava	800	200	Group A
	*L. borgpetersenii* serogroup Ballum serovar Ballum	100	<100	
Dog n. 2	*L. interrogans* serogroup Icterohaemorragiae serovar Copenhageni	400	<100	
	*L. interrogans* serogroup Australis serovar Bratislava	100	<100	Group A
Dog n. 3	*L. interrogans* serogroup Icterohaemorragiae serovar Copenhageni	200	200	Group A
Dog n. 4	*L. interrogans* serogroup Icterohaemorragiae serovar Copenhageni	200	200	Group A
Dog n. 5	*L. interrogans* serogroup Icterohaemorragiae serovar Copenhageni	200	100	Group A
Dog n. 6	*L. interrogans* serogroup Icterohaemorragiae serovar Copenhageni	100	<100	Group B
	*L. interrogans* serogroup Australis serovar Bratislava	100	<100	
Dog n. 7	*L. interrogans* serogroup Icterohaemorragiae serovar Copenhageni	100	<100	Group B
Dog n. 8	*L. interrogans* serogroup Icterohaemorragiae serovar Copenhageni	1100	<100	Group B

**Table 4:** Results of multi-locus sequence typing (MLST) analysis.

Sample	ST	*glmU*	*pntA*	*sucA*	*tpiA*	*pfkB*	*mreA*	*caiB*
Puppy	17	1	1	2	2	10	4	8
Dog n. 1	17 (partial)	n.d.	1	2	2	10	n.d.	8

ST: sequence type; n.d.: not defined.

## Data Availability

The data presented in this study are available on request from the corresponding author.
